# Why Are Our Teeth Degenerating

**Published:** 1896-06

**Authors:** Arthur Turner

**Affiliations:** England.


ARTICLE III.

### WHY ARE OUR TEETH DEGENERATING.

ARTHUR TURNER, ENGLAND.

This is a question that is constantly being asked, but is not so easily answered. We cannot fix on any one source of mischief and say if this was removed the teeth of the race would return to the normal healthy condition of our ancestors.

The root of the mischief will, however, be found among the following causes:

First. Starvation, or scarcity of natural tissue-forming material in the food.

The Highlander who, still clings to the habits of his ancestors, eats his daily portion of oatmeal porridge and oatcake, knows comparatively little of caries till he forsakes the highlands and begins a city life. When he has fairly adopted the ordinary diet of the citizens his teeth show a tendency to decay, and he suffers. There is something in whole-grain foods which supplies stamina to the harder structures of the body. It is wanting in the winter breads which are unfortunately preferred by us.

Second. Overwork of the brain is undoubtedly another cause of tooth decay. If the brain be doing a normal amount of work it easily finds abundance of material in healthy blood to replenish it and repair its waste. But the brain that is in a constant condition of tension, worry and overstrain, tries to draw an overdraught of nourishing material from the blood supply. The blood thus impoverished cannot provide adequate nourishment for the other parts of the body, and the teeth suffer first, probably because even their normal blood-supply is small.

Third. Want of proper use of teeth and jaws. It is an almost universal rule that an organ not properly used will undergo a process of degeneration and decay. The daintier foods of civilized life do not require the teeth to do the hard work as the fare of our ancestors did. Again, if one hurries over a meal it is impossible even to give modern foods the amount of mastication they ought to have. Hence our teeth are called on to exert a gradually decreasing amount of grinding power, and in the course of generations less power is provided. The jaws are getting smaller and smaller. The wisdom tooth is disappearing altogether. The other teeth are becoming less efficient because apparently less needed.

Fourth. Of all causes of decay, uncleanliness is perhaps the most fertile. The clean tooth may decay—the neglected tooth must decay. It has lost its chance of self-defense.

Decay is caused by minute forms of life. If the teeth do not have their necessary daily cleansing these germs multiply in the debris of food and saliva, especially at the gum border. Another source of mischief is the acidity of food and saliva, especially at the gum border. Another source of mischief is the acidity of many unhealthy mouths.

Our cleansing material should contain some efficient and pleasant antiseptic. It must also contain a proportion of some harmless and agreeable antacid to neutralize acidity.

The use of the tooth-brush was not so necessary to our ancestors because their coarser foods and more powerful mastication served in a great measure to keep the surface of the teeth clean without artificial aid.

These then are the principal causes of the degeneration of our teeth. If we sum them up we shall find they are all due to the tendencies of civilization to lead us away from the natural life of our ancestors.

When ought we go to the dentist? Many think it unnecessary to devote particular attention to the teeth till the appearance of the mouth is affected by damaged, decayed, or broken teeth. Others give their teeth no attention till pain compels them to do so. The stupidity and short-sightedness of either policy is evident. Every one who thinks a moment on the subject knows that we cannot masticate our food satisfactorily if one or more of the teeth are tender, inflamed, decayed, or otherwise out of working order, and that if this be the case the mouth requires immediate attention.

Here let us say a word or two on the importance of mastication. The process of digestion begins in the mouth. Trituration and mastication are voluntary acts, and are liable to be neglected unless some attention be given to the subject, and a definite habit formed.

The best codes of rules for athletic training insist on every mouthful of food being thoroughly and carefully masticated. If this be neglected the nutriment from the food cannot be transferred to the blood in proper proportion and the tissues cannot be properly repaired and built up. Take away proper mastication, and you take away this repair; remove the possibility of this repair and you must in time remove health, vigor, and all that makes physical life worth the living. Hence the importance of having the mouth in thoroughly good working order, either with sound or repaired natural teeth, or artificial substitutes.—*Items of Interest.*

